# Illuminating the Off-Pathway Nature of the Molten Globule Folding Intermediate of an α-β Parallel Protein

**DOI:** 10.1371/journal.pone.0045746

**Published:** 2012-09-21

**Authors:** Simon Lindhoud, Adrie H. Westphal, Jan Willem Borst, Carlo P. M. van Mierlo

**Affiliations:** 1 Laboratory of Biochemistry, Wageningen University, Wageningen, The Netherlands; 2 Microspectroscopy Centre, Wageningen University, Wageningen, The Netherlands; Uni. of South Florida, United States of America

## Abstract

Partially folded protein species transiently form during folding of most proteins. Often, these species are molten globules, which may be on- or off-pathway to the native state. Molten globules are ensembles of interconverting protein conformers that have a substantial amount of secondary structure, but lack virtually all tertiary side-chain packing characteristics of natively folded proteins. Due to solvent-exposed hydrophobic groups, molten globules are prone to aggregation, which can have detrimental effects on organisms. The molten globule observed during folding of the 179-residue apoflavodoxin from *Azotobacter vinelandii* is off-pathway, as it has to unfold before native protein can form. Here, we study folding of apoflavodoxin and characterize its molten globule using fluorescence spectroscopy and Förster Resonance Energy Transfer (FRET). Apoflavodoxin is site-specifically labeled with fluorescent donor and acceptor dyes, utilizing dye-inaccessibility of Cys69 in cofactor-bound protein. Donor (i.e., Alexa Fluor 488) is covalently attached to Cys69 in all apoflavodoxin variants used. Acceptor (i.e., Alexa Fluor 568) is coupled to Cys1, Cys131 and Cys178, respectively. Our FRET data show that apoflavodoxin’s molten globule forms in a non-cooperative manner and that its N-terminal 69 residues fold last. In addition, striking conformational differences between molten globule and native protein are revealed, because the inter-label distances sampled in the 111-residue C-terminal segment of the molten globule are shorter than observed for native apoflavodoxin. Thus, FRET sheds light on the off-pathway nature of the molten globule during folding of an α-β parallel protein.

## Introduction

Folding of proteins to conformations with proper biological activities is of vital importance for all living organisms. To describe protein folding, the concept of a multidimensional energy landscape or folding funnel arose from a combination of experimental data, theory and simulation [1], [2], [3], [4], [5]. In this model, proteins descend along a funnel wall describing the free energy of folding, until they reach the native state. Folding energy landscapes usually are rugged and comprise kinetic traps and barriers that pose restrictions on the way to the native state. As a result, partially folded intermediates are formed, which may be on- or off-pathway to the native state. When the intermediate is on-pathway, as is observed for the majority of proteins studied to date, it has native-like topology and is productive for folding. In contrast, when the intermediate is off-pathway, it is trapped in such a manner that the native state cannot be reached without substantial reorganizational events [6].

The resemblance between early kinetic intermediates and molten globules [7], [8], [9], [10] suggests that these molten globules can be considered as models of transient intermediates [6]. Several kinetic studies have revealed involvement of off-pathway intermediates during protein folding (see e.g. [11], [12], [13]). The formation of a kinetically trapped off-pathway molten globule increases the likelihood of protein aggregation. Elucidation of the formation and conformation of molten globules offers potential insights into factors responsible for protein misfolding, aggregation, and, potentially, for numerous devastating pathologies [14], [15].

Structural characterization of molten globules is hampered by the often transient nature of their existence, their usually relatively low population at equilibrium, and their aggregation at high protein concentrations [16]. One needs techniques that detect these species with high sensitivity, and thus fluorescence spectroscopy and the phenomenon of Förster Resonance Energy Transfer are very suitable [17], [18], [19]. FRET is the distance dependent transfer of electronic excitation energy from a donor fluorophore to an acceptor chromophore through nonradiative dipole-dipole coupling. This phenomenon enables detection of distances between donor and acceptor molecules of typically <10 nm [17], [20], [21]. The FRET efficiency (*E*) strongly depends on the distance (*r*) between a donor and an acceptor molecule, according to:
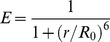
(1)with *R_0_* being the Förster distance, i.e., the distance at which the energy transfer efficiency is 50%. Labeling of proteins with bright donor and acceptor dyes strongly facilitates the use of FRET to study protein folding. Here, we employ FRET to study the folding of dye-labeled *A. vinelandii* apoflavodoxin, and to characterize its off-pathway molten globule.

Flavodoxins are monomeric flavoproteins that are involved in electron transfer and contain a non-covalently bound FMN cofactor [22]. These proteins adopt the flavodoxin-like topology, also referred to as the doubly-wound or α-β parallel topology. This topology is among the most common topologies in the protein databank, and is shared by many functionally and sequentially unrelated proteins.

We demonstrated previously that folding of apoflavodoxin occurs spontaneously. Time-dependent folding of apoflavodoxin involves an energy landscape with two intermediates, and is described by the following kinetic model: I_off_ ⇔ unfolded apoflavodoxin ⇔ I_on_ ⇔ native apoflavodoxin [13], [23]. Non-covalent binding of FMN to native apoflavodoxin is the last step in flavodoxin folding [24]. Native apoflavodoxin (i.e., flavodoxin without FMN) strongly resembles flavodoxin, except for dynamic disorder in the flavin-binding region [25], [26]. Intermediate I_on_ lies on the productive folding route from unfolded to native protein and is highly unstable. Approximately 90% of folding molecules fold via off-pathway intermediate I_off_, which is a relatively stable species that needs to unfold to produce native protein and thus acts as a trap. Formation of an off-pathway folding species is probably inherent to the folding of proteins with a flavodoxin-like fold [27], as this species is experimentally observed for other α-β parallel proteins of which the kinetic folding has been investigated (i.e., apoflavodoxin from *Anabaena* [11], CheY [28], cutinase [29], and UMP/CMP kinase [30]).

**Figure 1:**
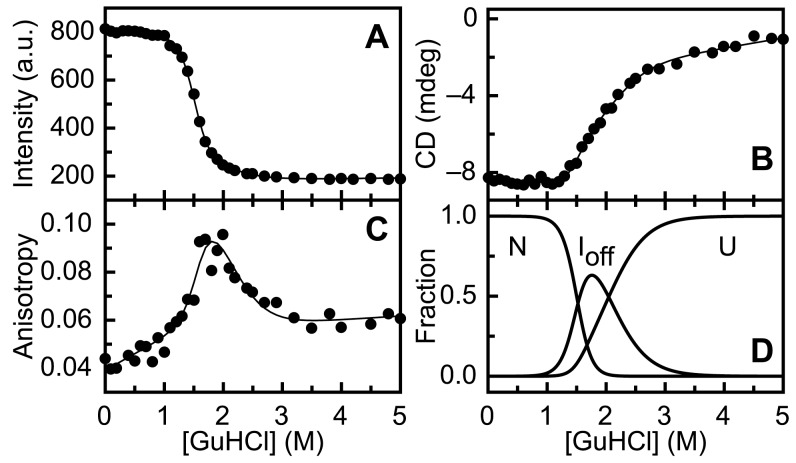
Denaturant-dependent equilibrium folding shows involvement of an intermediate during apoflavodoxin folding, as demonstrated by [**13**] **.** (a) Fluorescence emission intensity of tryptophan detected at 340 nm upon excitation at 280 nm. (b) CD signal at 222 nm. (c) Fluorescence anisotropy data detected with a 335 nm cut-off filter, excitation is at 300 nm. Solid lines in panels a to c are the result of a global fit of a three-state model for equilibrium (un)folding. (d) Normalized population of native (N), off-pathway intermediate (I_off_), and unfolded (U) apoflavodoxin molecules as a function of denaturant concentration. Protein concentration is 5.6 µM in 100 mM potassium pyrophosphate, pH 6.0, and data are recorded at 25°C.

During denaturant-induced equilibrium unfolding, denaturant is added to apoflavodoxin and subsequently the protein is allowed to reach thermodynamic equilibrium before acquisition of spectroscopic data. Figure 1a shows that the guanidine hydrochloride (GuHCl) concentration at the midpoint of denaturation of apoflavodoxin as detected by fluorescence spectroscopy is 1.533±0.007 M. The midpoint of denaturation as detected by far-UV CD in Fig. 1B is 1.72±0.05 M GuHCl. Thus, the folding curves obtained by CD and fluorescence spectroscopy do not coincide. This non-coincidence is a characteristic for population of a folding intermediate state. Furthermore, fluorescence anisotropy data of apoflavodoxin depend biphasically on denaturant concentration (Fig. 1c), which is another characteristic for population of a folding intermediate state. Hence, description of apoflavodoxin equilibrium folding data requires use of a model that takes native, intermediate and unfolded states into account. Because intermediate I_on_ is highly unstable, this intermediate is not detected at equilibrium. Consequently, denaturant-dependent equilibrium folding of apoflavodoxin is described by the three-state equilibrium model I_off_ ⇔ unfolded apoflavodoxin ⇔ native apoflavodoxin (Fig. 1) [13]. Off-pathway intermediate I_off_ populates significantly at equilibrium, enabling characterization of its properties. For example, at about 2 M GuHCl virtually no native apoflavodoxin molecules are present as judged from fluorescence data (Fig. 1a), but still a significant CD signal is observed (Fig. 1b). Thus, the folding intermediate has a substantial amount of secondary structure, but lacks the tertiary side-chain packing of natively folded apoflavodoxin. This observation is typical for a molten globule-like intermediate. Apoflavodoxin’s molten globule species is compact, its three tryptophans are solvent-exposed, and it has severely broadened NMR resonances due to exchange between different conformers on the micro- to millisecond time scale [13], [31], [32], [33]. Elevated apoflavodoxin concentrations and molecular crowding cause severe aggregation of this molten globule [32], [33].

Unfolded apoflavodoxin has four transiently ordered regions. Three of these regions (i.e., Ala41-Gly53, Gln99-Ala122, and Thr160-Gly176) transiently form α-helices and the fourth region (i.e., Glu72-Gly83)) transiently adopts non-native structure, which is neither α-helix nor β-strand [34]. Upon reducing denaturant concentration, the four structured elements in unfolded apoflavodoxin transiently interact and subsequently form the ordered core of the molten globule [34], [35], [36], [37], [38].

In this study, we monitor denaturant-dependent equilibrium folding of doubly dye-labeled apoflavodoxin variants using ensemble fluorescence and FRET. To obtain these proteins, we introduce cysteine residues at appropriate positions into apoflavodoxin. In addition, site-specific labeling with equimolar ratio of donor to acceptor is desired [21], [39], because fluorescence emissions of labels are differently affected by their corresponding local environment. To fulfill this criterion, we utilize specific properties of the cofactor-bound form of the protein. Donor (i.e., Alexa Fluor 488 C_5_ maleimide; A488) is coupled to Cys69, whereas acceptor (i.e., Alexa Fluor 568 C_5_ maleimide; A568) is attached to Cys1, Cys131, or Cys178, respectively. These dyes are brightly fluorescent, photostable, and excitable by visible light [40], [41]. The corresponding doubly dye-labeled proteins are called d69-a1, d69-a131, and d69-a178, respectively. Subsequent measurements of fluorescence emission and FRET during apoflavodoxin folding reveal hitherto unknown features of the molten globule folding intermediate of an α-β parallel protein.

## Results

### Site-specific Dye-labeling to Track Protein Folding

By site-directed mutagenesis, we designed apoflavodoxin variants A001C, D131C, and S178C, respectively. The introduced cysteines reside in solvent-accessible loops (Fig. S1), thus enabling dye labeling. All protein variants expressed in *E. coli* contain tightly bound FMN. Tight binding of FMN occurs primarily through a very specific combination and geometry of hydrogen bonds and aromatic interactions with native apoflavodoxin. Consequently, the observation that all apoflavodoxin variants of this study bind FMN tightly implies that the three-dimensional structures of the corresponding flavodoxins are nearly indistinguishable from the one of wild-type flavodoxin. Analogously, the conformations of native apoflavodoxin variants thus are similar.

The introduced cysteines are more accessible than Cys69 (Fig. S1), allowing site-specific labeling with A568. Under the given experimental conditions hardly any labeling of Cys69 takes place (Fig. S2). Subsequent unfolding of each acceptor-labeled variant enables labeling of Cys69 with A488. The degree of doubly dye-labeling is similar, because the absorption spectra of the proteins almost completely overlap (Fig. S3a). Equimolar ratio of donor to acceptor is obtained.

Upon removal of denaturant, GuHCl-unfolded, dye-labeled apoflavodoxin variants autonomously fold to native apoprotein, because apoflavodoxin unfolding is reversible [31]. Subsequent addition of FMN leads to complete reconstitution of holoprotein (data not shown), and thus, coupling of donor and acceptor dyes does not impede the ability of apoflavodoxin to bind cofactor.

**Figure 2:**
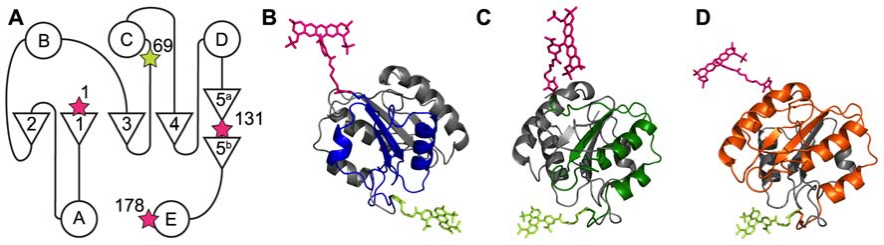
Covalent attachment of dye-labels to enable FRET-based probing of folding of various regions of apoflavodoxin. In all protein variants, donor label (i.e., A488) is attached to residue 69. (a) Positions of dye labels within the topology of flavodoxin. A488 is represented by a bright green star and acceptor label (i.e., A568) by a pink star. (b) Cartoon representation of d69-a1, showing in blue the backbone that intervenes residues 1 and 69. (c) d69-a131, with the backbone intervening residues 69 and 131 in green. (d) d69-a178, with the backbone intervening residues 69 and 178 in orange. A488 is shown in bright green, and A568 in pink. Cartoons are generated with PyMOL (Schrödinger, LLC, Palo Alto, Ca, USA) using the crystal structure of *A. vinelandii* flavodoxin (pdb ID 1YOB [56]) and the molecular structures of A488 and A568, as provided by Invitrogen.

Use of d69-a1 allows tracking of the folding of the N-terminal part of the protein, which consists of α-strands 1, 2 and 3, and α-helices A and B of native apoflavodoxin (Figs. 2a, b). Use of d69-a131 reports about the folding of residue 69 up to and including residue 131, consisting of β-strands 4 and 5a, and α-helices C and D of native apoflavodoxin (Figs. 2a, c), and use of d69-a178 additionally informs about the folding of residues 131 up to and including residue 178, consisting of β-strand 5b and C-terminal α-helix E of native protein (Figs. 2a, d).

### Folding of Dye-labeled Apoflavodoxins Involves a Stable Intermediate

**Figure 3:**
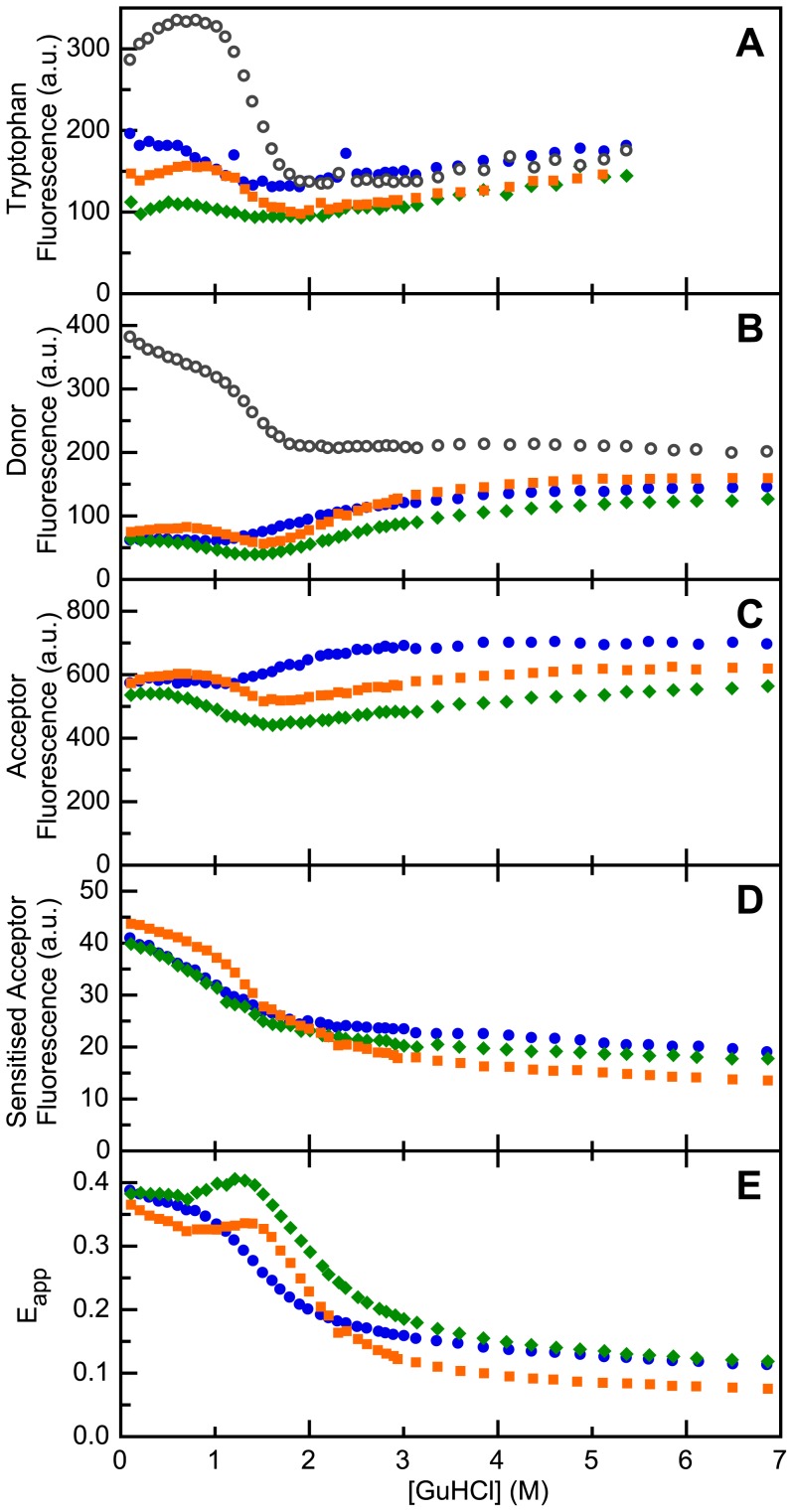
The denaturant-dependence of fluorescence signals of doubly dye-labeled apoflavodoxin reveals properties of apoflavodoxin’s folding intermediate. Shown are fluorescence data of ‘donor-only’ protein (open circles), d69-a1 (blue circles), d69-a131 (green diamonds) and of d69-a178 (orange squares). Protein concentration is 62.5 nM. (a) Fluorescence emission intensity of tryptophan at 330 nm with excitation at 280 nm. (b) Fluorescence emission intensity of donor (A488) at 515 nm with excitation at 450 nm. (c) Fluorescence emission intensity of acceptor (A568) at 630 nm with excitation at 580 nm. (d) Sensitized fluorescence emission intensity of acceptor at 630 nm with excitation at 450 nm. (e) Apparent FRET efficiency (*E_app_*), calculated by using data of panels (b) and (d) and equation 3. Standard deviations of the fluorescence signals shown vary between 1 to 3% of the measured signal intensities.

Participation of a folding intermediate is observed during folding of apoflavodoxin that is labeled with a donor and an acceptor label, as the following data show. Figures 3a to 3c present fluorescence emission intensities of tryptophan, A488, and of A568 during denaturant-dependent equilibrium folding of doubly dye-labeled protein. Fluorescence emission intensities of donor (i.e., A488 (Fig. 3b)) and of acceptor (i.e., A568 (Fig. 3c)) reports biphasic unfolding curves for d69-a131 and d69-a178. This biphasic behavior demonstrates population of a stable intermediate during folding of both proteins.

Compared to native and unfolded protein, the intermediate has lower fluorescence emission intensities of donor as well as of acceptor. Use of FRET enables further characterization of this intermediate, as discussed below. Unfolding curves of d69-a1 show no clear biphasic pattern (Figs. 3b,c). Nevertheless, a stable intermediate populates during its folding, because the transition revealed by fluorescence emission intensities of tryptophans (Fig. 3a) and the transitions revealed by fluorescence emission intensities of A488 and A568 do not coincide. This non-coincidence of unfolding transitions is a hallmark of the involvement of a folding intermediate. In conclusion, folding of all three doubly dye-labeled apoflavodoxin variants involves a stable folding intermediate.

Fluorescence of tryptophans of doubly dye-labeled apoflavodoxin has a complex dependency on denaturant due to FRET between the three tryptophan residues of apoflavodoxin (i.e., Trp74, Trp128, and Trp167) [42], and because FRET occurs between tryptophans and donor as well as acceptor labels (see below). Similarly, FRET between donor and acceptor labels influences fluorescence emission of donor as a function of denaturant concentration. Due to the complex dependence of fluorescence intensity on denaturant concentration when FRET is occurring, and because this dependence is not quantitatively predictable owing to the strong distance dependence of fluorescence intensity [43], [44], we can not use fluorescence signals to calculate thermodynamic parameters of doubly dye-labeled native proteins and corresponding folding intermediates. Attachment of dye labels to apoflavodoxin will have some impact on the thermodynamic stabilities of the different folding species. Indeed, comparison of Figs. 1a and 3a shows that coupling of donor and acceptor to apoflavodoxin destabilizes native apoprotein, because the corresponding folding transition shifts to lower concentration GuHCl. Similarly, upon mutating various amino acid residues of apoflavodoxin, stabilities of the corresponding native apoproteins decrease [31], [37], [38], [45]. Yet, just as observed here for doubly dye labeled protein, folding still occurs according to a three-state model, because this is a typical feature of proteins with a flavodoxin-like fold [27].

### Fluorescence of Dye-labeled Apoflavodoxin in the Native and Unfolded State

Due to FRET, tryptophan fluorescence of native protein decreases considerably upon covalent attachment of A488 to Cys69 (*R_0_*∼27 Å, as calculated using the information provided in Fig. S3b). Upon subsequent covalent coupling of A568 to ‘donor-only’ apoflavodoxins, tryptophan fluorescence of native protein diminishes even further (Fig. 3a), because A568 functions as additional acceptor for FRET (*R_0_*∼26 Å, as calculated using the information provided in Fig. S3b). To obtain rough estimates of the separations between tryptophans and dye labels, we determined distances between C^α^ of dye-labeled residues and C^7a^ of tryptophan residues (Table 1). The distance between C^α^ of residue 131 and the most nearby tryptophan (i.e., C^7a^ of Trp128) is 8.7 Å, whereas the distance between C^α^ of residue 1 and its most nearby tryptophan (i.e., C^7a^ of Trp167) is 18.3 Å. In case of d69-a178, 14.8 Å separates C^α^ of residue 178 and its most nearby tryptophan (i.e., C^7a^ of Trp167). Correspondingly, in their native states, d69-a131 has the lowest tryptophan fluorescence, whereas d69-a1 has the highest emission (Fig. 3a).

**Table 1:** Distances between residues used in dye-labeling and tryptophans of native apoflavodoxin.

From	To		
	Trp74	Trp128	Trp167
Cys1	21.3 Å	24.3 Å	18.3 Å
Cys69	12.8 Å	26.4 Å	24.5 Å
Cys131	25.3 Å	8.7 Å	15.2 Å
Cys178	26.1 Å	19.3 Å	14.8 Å

The distances reported are between C^7a^ of the tryptophan indicated and C^α^ of the residue to be dye-labeled, as measured by using PyMol (Schrödinger, LLC, Palo Alto, Ca, USA) and the crystal structure of *A. vinelandii* flavodoxin (pdb ID 1YOB [56]).

Tryptophan fluorescence data show that the unfolded baselines of the folding curves of ‘donor-only’ apoflavodoxin and d69-a1 coincide. Hence, in unfolded protein no FRET from tryptophans to A568 occurs, as apparently their spatial separation exceeds twice the corresponding Förster distance. In case of unfolded d69-a131 and d69-a178, FRET plays a role, because their tryptophan fluorescence is lower than that of ‘donor-only’ apoflavodoxin (Fig. 3a). FRET from tryptophans to acceptor is similar in these unfolded proteins, because their unfolded baselines coincide.

To quantify FRET between A488 and A568 during denaturant-dependent equilibrium folding of doubly dye-labeled apoflavodoxin, we use equation 2:
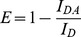
(2)where *I_DA_* and *I_D_* are the fluorescence emission intensities of donor label in presence and in absence of acceptor, respectively. We obtained *I_D_* by measuring the denaturant dependency of fluorescence emission of ‘donor-only’ protein (i.e., d69-apoflavodoxin) at 515 nm, which appears to track folding of this protein (Fig. 3b). Upon attaching acceptor label to native ‘donor-only’ apoflavodoxin, fluorescence intensity of donor A488 severely decreases, as comparison of native baselines in Fig. 3b shows. This decrease is due to FRET from A488 to A568. Whereas at zero molar denaturant this FRET between both dyes is similarly efficient for d69-a1 and d69-a131, it is less efficient in case of d69-a178. Upon unfolding of doubly dye-labeled protein above 2 M GuHCl, donor fluorescence increases and levels off at high concentrations of denaturant (Fig. 3b). Fluorescence emission intensity of A488 of fully unfolded protein at 6.9 M GuHCl decreases from d69-a178, d69-a1 to d69-a131. This order is consistent with the number of amino acid residues in between donor and acceptor in these proteins, which decreases from 108, to 67, and to 61 residues, respectively.

Besides diminishing of tryptophan fluorescence due to transfer of excitation energy to Alexa dyes, we observe folding-dependent quenching of fluorescence of directly excited acceptor (Fig. 3c). Similar changes also happen for A488 fluorescence in case of ‘donor-only’ apoflavodoxin (Fig. 3b). Recently, tryptophan has been identified as the most potent photon induced electron transfer quencher of Alexa fluorophores. When such a fluorophore is in proximity of tryptophan, photon induced electron transfer towards tryptophan can occur and as a result fluorescence of the dye involved diminishes due to static quenching [46], [47]. Due to this quenching, A568 fluorescence of unfolded apoflavodoxin decreases from d69-a1, to d69-a178, and to d69-a131, respectively. This order reflects the number of amino acid residues in between acceptor and nearest tryptophan in the primary sequence of the protein (i.e., 72, 10, and 2 residues, respectively). In case of native protein, Trp74 and Trp167 are in the protein interior, whereas Trp128 is located at the protein surface [42]. Consequently, in native protein, quenching of acceptor in d69-a131 is more efficient than in case of the other two doubly dye-labeled apoflavodoxins, for which A568 is about equally fluorescent (Fig. 3c).

Changes in quenching upon formation of apoflavodoxin’s molten globule cause the dip in fluorescence emission intensity of A568 of d69-a131, and of d69-a178 at ∼1.7 M GuHCl (Fig. 3c). Upon formation of this intermediate, tryptophans become solvent exposed [13], [38], which leads to increased quenching of A568. Remarkably, in case of d69-a1, Fig. 3c shows that A568 fluorescence rises upon increasing denaturant concentration. This observation reveals molecular details of apoflavodoxin’s folding intermediate, as shown below.

### FRET Tracks Folding of Dye-labeled Apoflavodoxin

By using fluorescence of donor label in presence and in absence of acceptor (i.e., *I_DA_* and *I_D_*, respectively (Fig. 3b)), and subsequent application of equation 2, we determine FRET efficiencies (*E*) of native and unfolded doubly dye-labeled apoflavodoxins. Table 2 reports these values for native protein at 0.1 M GuHCl (i.e., at the lowest concentration denaturant used) and for unfolded protein at 6.9 M GuHCl, at which apoflavodoxin behaves as a random coil [34]. FRET efficiencies range from 0.80 to 0.83 for native doubly dye-labeled apoflavodoxins. Using *R_0_* of 53.1±0.5 [33], we calculate that the corresponding inter-dye distances range from 40±2 to 42±2 Å, which is in reasonable agreement with the molecular dimensions of native apoflavodoxin. Unfolded apoflavodoxins are characterized by much lower FRET efficiencies, as the average distances between donor and acceptor labels are increased compared to the distances in native protein. FRET efficiencies range from 0.20, to 0.27, to 0.37, for unfolded d69-a178, unfolded d69-a1, and for unfolded d69-a131, respectively. This increase correlates with the inter-residue separations of both dyes in the primary sequences of these unfolded proteins.

**Table 2:** FRET efficiencies of native and unfolded doubly dye-labeled apoflavodoxins.

Protein variant	Fluorescence of nativeprotein (a.u.)	FRET efficiency of native protein	Fluorescence of unfolded protein (a.u.)	FRET efficiency of unfolded protein
d69	382.15±5.17		201.69±3.18	
d69-a1	62.84±1.85	0.84±0.03	145.99±2.55	0.27±0.01
d69-a131	63.45±1.93	0.83±0.03	126.86±2.88	0.37±0.01
d69-a178	74.89±2.61	0.80±0.03	159.51±2.49	0.20±0.01

Fluorescence emission intensity is of donor (A488) at 515 nm with excitation at 450 nm. Fluorescence of native protein is determined at 0.1 M GuHCl (i.e., at the lowest concentration denaturant used), whereas fluorescence of unfolded protein is determined at 6.9 M GuHCl.

The doubly dye-labeled apoflavodoxins enable monitoring of FRET efficiencies during folding. Attachment of acceptor label to ‘donor-only’ protein affects the thermodynamic stabilities of apoflavodoxin’s folding species. Consequently, in the transition regions of unfolding of ‘donor-only’ and doubly dye-labeled proteins the populations of these species differ. As a result, donor fluorescence of ‘donor-only’ protein (i.e., *I_D_*) and of doubly dye-labeled protein (i.e., *I_DA_*) cannot be used to determine the exact FRET efficiencies of doubly dye-labeled apoflavodoxin species according to equation 2. Therefore, we utilized the measured donor fluorescence intensities of doubly dye-labeled protein, and acquired the emission of acceptor upon excitation of donor, to calculate the apparent FRET efficiency (*E_app_*) at various denaturant concentrations, according to:
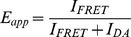
(3)where *I_FRET_* is the sensitized fluorescence emission intensity of acceptor (i.e., emission of acceptor upon excitation of donor). With identical fluorescence quantum yields of donor and acceptor and identical detection efficiencies of both fluorescence signals, *E_app_* equals *E*.

Figure 3d shows the denaturant-dependence of *I_FRET_* (i.e., fluorescence emission intensity of acceptor A568 at 630 nm with excitation of donor A488 at 450 nm) for all doubly dye-labeled apoflavodoxins. Application of equation 3 to the data of Figs. 3b and 3d yields Fig. 3e, which shows the corresponding *E_app_*. Clearly, because the *E_app_*-values are significantly lower than the corresponding *E*-values mentioned previously, considerable differences between fluorophore quantum yields and/or detection efficiencies of donor and acceptor dyes exist, causing downscaling of *E_app_* compared to *E*. Indeed, fluorescence quantum yields of A488 and A568 are 0.84 and 0.63, respectively (Invitrogen). In addition, whereas we measure fluorescence emission of A488 at its emission maximum of 515 nm, we acquire fluorescence emission of A568 at 630 nm, to avoid simultaneous detection of donor fluorescence. Fluorescence intensity of A568 at 630 nm is only ∼50% of its emission maximum, and thus *E_app_* becomes even further reduced.

Changes in *E_app_* track folding of dye-labeled apoflavodoxin (Fig. 3e). In case of d69-a131 and d69-a178, a hump in the corresponding unfolding curves highlights the presence of a folding intermediate. Remarkably, despite that folding of d69-a1 involves a stable intermediate, as discussed, no such hump is observed for this protein (Fig. 3e).

### Dependence of Förster Distance on Folding State

To further assess the molecular source of the hump in the unfolding curve of d69-a131 and of d69-a178 (Fig. 3e), one needs to address the effects of changing from one folding state to another has on the parameters that comprise the Förster equation:
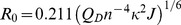
(4)where *Q_D_* is the quantum yield of donor fluorescence in absence of acceptor, *n* the refractive index of the medium that separates donor from acceptor [48], *κ* ^2^ the orientation factor for the relevant transition dipole moments, and *J* the integrated spectral overlap of acceptor absorbance and donor fluorescence spectra (M^−1^ cm^3^).

Upon changing the folding state, *Q_D_* in equation 4 possibly alters. Figure 3b suggests that native ‘donor-only’ apoflavodoxin has a *Q_D_*-value higher than that of the corresponding unfolded protein or folding intermediate. Consequently, these latter protein states would have *R_0_*-values that are lower than the one associated with native protein. For example, decreasing *Q_D_* from 0.84 to 0.5 results in a drop of *R_0_* from 53.1 Å to 48.7 Å [33]. In the hypothetical situation that the distance between donor and acceptor dyes remains unaltered upon switching folding states, but *Q_D_* diminishes, both folding intermediate and unfolded protein would show less FRET than native protein. However, Fig. 3e reports increased FRET for folding intermediate compared to FRET for native and unfolded protein.

The refractive index of the medium that separates donor from acceptor alters by changing denaturant concentration and thus *n* in equation 4 changes. With refractometry we determined that the refractive index of buffer (i.e., 100 mM KPPi, pH 6.0) is 1.337, whereas the refractive index of buffer with 6.9 M GuHCl is 1.451. The interior of native protein has a refractive index of ∼1.6 [49], and consequently the refractive index of protein lies somewhere between approximately 1.3 and 1.6, since the medium is a mixture of buffer, GuHCl and protein. A reasonable estimate for the refractive index of hydrated protein (∼50% protein and ∼50% water), which separates donor from acceptor label in apoflavodoxin, is a value of ∼1.5 [48], [50]. Upon adding denaturant, *n* slightly increases and thus *R_0_* decreases. Consequently, again in the hypothetical situation that donor and acceptor dyes would remain fixed upon switching folding states, both folding intermediate and unfolded protein would give rise to slightly less FRET than native protein. Figure 3e clearly shows that this situation is not the case for folding intermediate.

Assessing the effects a change of folding state has on *κ^2^*, and thus on *R_0_*, is difficult to achieve. The dipole orientation factor *κ^2^* equals:
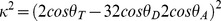
(5)in which *θ_T_* represents the angle between donor emission dipole and acceptor absorption dipole, and *θ_D_* and *θ_A_* are the angles between these dipoles and the vector that connects donor and acceptor. In case of donor and acceptor pairs having unrestricted flexibility, *κ^2^* equals 2/3.

Despite that the dyes are attached to apoflavodoxin via flexible linkers, dye-reorientation might be restricted by interactions of the fluorophores with the protein surface. These interactions may differ between folding states. To assess *κ^2^* associated with these states, we measured time-resolved fluorescence anisotropy to determine the reorientation rates of A488 and A568, using ‘donor-only’ and doubly dye-labeled protein at increasing concentrations denaturant (see Materials and Methods). The anisotropy decay (*r_t_*) of a dye-labeled native protein exhibiting slow overall rotation with time constant *Φ_prot_* and amplitude *β_2_*, and fast reorientation of the attached dye with time constant for internal reorientation *Φ_dye_* and amplitude *β_1_*, is described by the following model [51]:
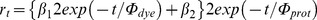
(6)where the sum of the amplitudes is the fundamental anisotropy (i.e., *r_0_* = *r_t_*(t = 0)). This model excellently describes anisotropy decay of dye-labeled apoflavodoxin at each concentration of denaturant used (Fig. S4). Note that upon unfolding, overall rotation of the protein decreases, while concomitantly flexibility of the polypeptide increases considerably. As a result, for unfolded protein, *Φ_prot_* reports the combined local dynamics of the label and of the amino acid to which the dye is covalently coupled. Taking the measured anisotropy data one can calculate a second-rank order parameter *S* for the reorienting fluorophores that are attached to apoflavodoxin, according to [51]:



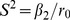
(7)Thus, when the dye does not reorient with respect to protein (i.e., dye motion is fully restricted) *S* equals 1. The rate of dye reorientation is given by the diffusion coefficient *D*_⊥_ of internal motion, which is calculated according to [51]:
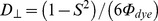
(8)


**Figure 4:**
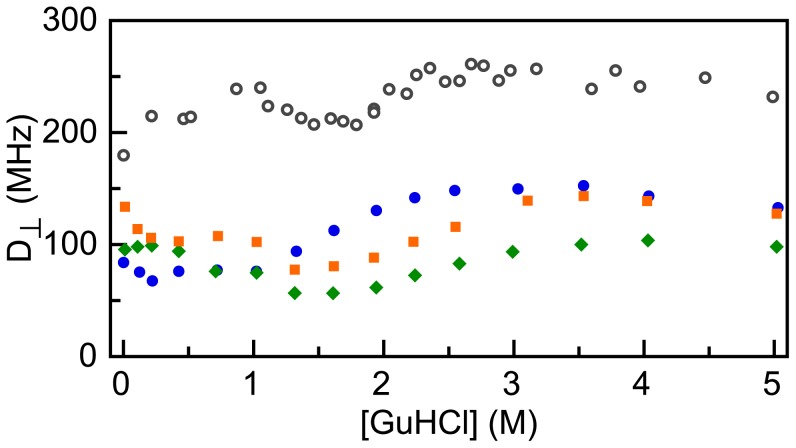
Denaturant-dependencies of the reorientation rates of dye labels attached to apoflavodoxins. Shown are the *D*_⊥_ data of A488 of ‘donor-only’ protein (open circles), and of A568 of d69-a1 (blue circles), d69-a131 (green diamonds) and d69-a178 (orange squares), respectively.

Figure 4 shows the rates of dye reorientation of A488 in ‘donor-only’ apoflavodoxin, as well as of A568 in doubly dye-labeled apoflavodoxin variants. The value of *D*_⊥_ for all denaturant concentrations used is well above 50 MHz (Fig. 4) and shows that the dye labels exhibit flexibility. Consequently, during our measurements, donor emission and acceptor excitation dipoles are randomly oriented towards one another, which justifies the assumption that *κ* ^2^ = 2/3 at all denaturant concentrations used.

**Figure 5:**
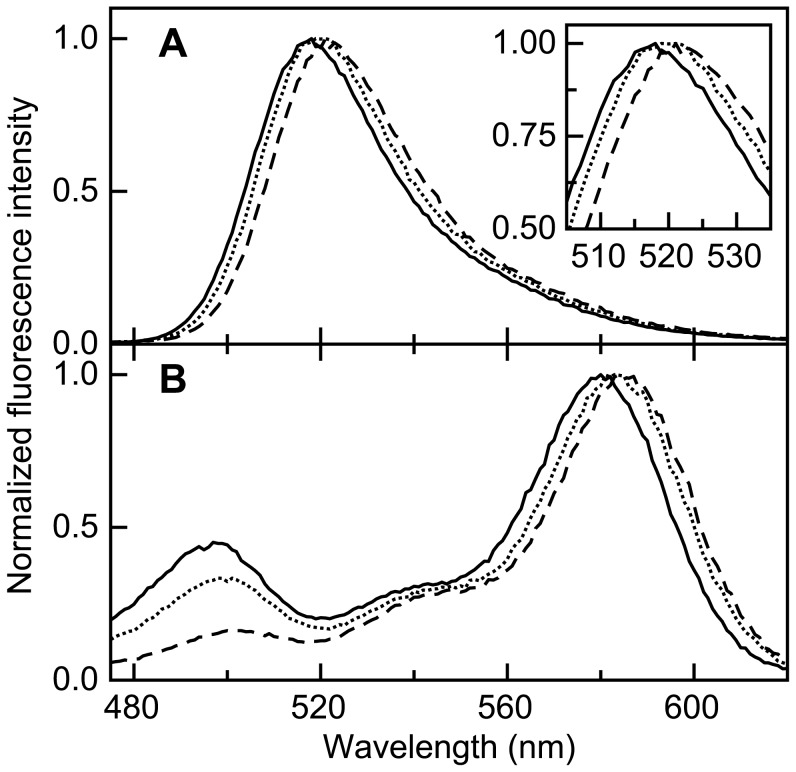
Upon adding denaturant, apoflavodoxin’s donor emission spectrum and acceptor excitation spectrum shift to the red. (a) Normalized emission spectra of ‘donor-only’ apoflavodoxin. The inset zooms in on the fluorescence emission maximum, which shifts from 518 to 521 nm upon adding 6.9 M GuHCl. (b) Normalized excitation spectrum of acceptor of d69-a178. GuHCl concentrations are 0.1 M (solid line), 1.7 M (dotted line) and 6.9 M (dashed line), respectively.

Finally, upon changing folding state, the spectral overlap integral *J* of the Förster equation might alter. This integral equals:
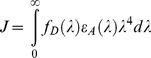
(9)where *f_D_*(*λ*) is the normalized fluorescence emission spectrum of donor, and *ε_A_*(*λ*) is the normalized absorption spectrum of acceptor weighted by the corresponding molar extinction coefficient. Figure 5 shows that upon adding 6.9 M GuHCl to apoflavodoxin, both the emission spectrum of donor as well as the excitation spectrum of acceptor shift to the red by about 3 to 4 nm, and concomitantly *ε_A_* increases by about 11%. This combined effect causes a 14% increase in the integrated spectral overlap and thus leads to a slightly larger Förster distance (i.e., *R_0_* increases 2.3% and changes from 53.1 to 54.3 Å).

In conclusion, upon switching between folding states, changes in parameters of the Förster equation are such that they cannot account for the observed hump in the *E_app_* unfolding curves of d69-a131 and d69-a178. These humps must thus reflect shortening of separation between donor and acceptor upon conversion of native protein to folding intermediate. Upon this transition, no such shortening happens in the N-terminal part of apoflavodoxin, because no accompanying hump exists in the *E_app_* unfolding curve of d69-a1.

## Discussion

Exploiting the dye-inaccessibility of Cys69 in cofactor-bound flavodoxin, we obtained a homogeneous population of doubly dye-labeled protein molecules with donor attached to Cys69 and acceptor at desired positions. In this study, we track folding of ensembles of site-specifically dye-labeled apoflavodoxin molecules by fluorescence emission intensities and apparent FRET efficiencies.

### Molecular Properties of Apoflavodoxin’s Off-pathway Folding Intermediate

Fluorescence emission intensity and *E_app_* data of the three doubly dye-labeled apoflavodoxins reveal hitherto unknown features of the off-pathway molten globule of apoflavodoxin. The hump observed in the *E_app_* data of denaturant-dependent folding of d69-a131 and d69-a178 (Fig. 3e) implies that donor and acceptor are less separated in the interconverting ensemble of conformers that represents *I_off_*, than they are in native apoflavodoxin. Concomitantly, fluorescence emission intensity of A568 drops (Fig. 3c), revealing exposure of hydrophobic side chains of tryptophans and tyrosines in folding intermediate, which both act as fluorescence quenchers of acceptor label (with tryptophan being the dominant quencher). Exposure of hydrophobic side chains probably causes a slight decrease in reorientation rate of A568, as Fig. 5 suggests. The C-terminal part of the molten globule, involving residues 69 to 178, must thus be rather compact and differs considerably from native protein. This part of the molten globule has a midpoint of unfolding of ∼2 M GuHCl.

### Folding of Apoflavodoxin’s Molten Globule is Non-cooperative

In case of d69-a1, where *E_app_* monitors folding of the N-terminal 69 residues of the protein, no hump exists in the corresponding denaturant-dependent folding curve (Fig. 3e). Despite the absence of this hump, apoflavodoxin’s folding intermediate does populate in between ∼0.8 to ∼3.0 M GuHCl, as the non-coincidence of the unfolding transitions of d69-a1 in Figures 3a-c demonstrates. Hence, the observed decrease of *E_app_* in this denaturant range implies that the N-terminal 69 residues of the folding intermediate unfold above 0.8 M GuHCl. This unfolding is supported by fluorescence emission intensity of A568, which also shows no hump during denaturant-dependent equilibrium folding of the protein (Fig. 3c). In native d69-a1, due to its vicinity and partial solvent accessibility, Tyr47 quenches fluorescence of A568. At about 1 M GuHCl, where folding intermediate is also present, this quenching sustains, implying that the N-terminal part of this intermediate must be structured at relatively low concentrations of GuHCl. Increasing denaturant concentration leads to increased fluorescence of A568, because the N-terminal part of the molten globule unfolds and because unfolded d69-a1 becomes populated. Unfolding of the N-terminal part of the molten globule thus occurs at lower denaturant concentration than happens for its C-terminal part. This observation shows that folding of apoflavodoxin’s molten globule is non-cooperative.

### Unfolding of Transiently Ordered Regions in Unfolded Apoflavodoxin

The data of Figure 3 imply that the unfolded state of apoflavodoxin, which is fully populated at about 3 M GuHCl, expands upon increasing denaturant concentration. Upon adding denaturant, *E_app_* diminishes for all three dye-labeled unfolded proteins (Fig. 3e), because the average separation between donor and acceptor dyes increases. In addition, fluorescence of A488 and A568 increases (Figs. 3b,c), due to less efficient energy transfer and because less quenching of both dyes by tryptophan and tyrosine residues occurs, as the average distances between these residues and dye labels increase. This expansion of unfolded protein with increasing denaturant reflects unfolding of transiently ordered regions that exist in unfolded apoflavodoxin at about 3 M GuHCl. At 6 M GuHCl, the protein behaves as random coil [34], [35].

### FRET Data Show that the Conformations of Molten Globule and Native Apoflavodoxin Differ Drastically

NMR spectroscopy shows that upon lowering denaturant concentration, structure formation within virtually all parts of unfolded apoflavodoxin precedes folding to the molten globule state. This folding transition is non-cooperative and involves a series of distinct transitions. Four structured elements in unfolded apoflavodoxin transiently interact and subsequently form the ordered core of the molten globule. This ordered core is gradually extended upon decreasing denaturant concentration [34], [35], [36], [37]. NMR spectroscopy detects formation of apoflavodoxin’s molten globule in an indirect manner through disappearance of resonances of unfolded protein. Resonances of the molten globule cannot be detected by this technique, because they are broadened beyond detection due to exchange between different conformers on the micro- to millisecond time scale [33], [35]. In contrast, in the study presented here we directly detect features of this molten globule by measuring fluorescence emission and FRET of the dye labels that are covalently attached to the protein. These fluorescence data show that upon decreasing denaturant concentration, the C-terminal 111 residues of the molten globule fold first, leading to a conformation that differs drastically from the one of the C-terminal part of native protein. The N-terminal part of this species is still unfolded and upon lowering denaturant concentration this protein part becomes structured, as the data of Figure 3 imply. This study shows that the conformations of molten globule and native protein differ considerably. Hence, to produce native α-β parallel protein, the molten globule needs to unfold, explaining why this folding species is off-pathway during folding of apoflavodoxin.

## Materials and Methods

### Protein Engineering, Expression and Purification of Flavodoxin Variants Containing Cysteine Pairs

Single oligonucleotide site-directed mutagenesis [52] was used to generate three variants of *Azotobacter vinelandii* (strain ATCC 478) flavodoxin II, which each contain a pair of cysteine residues. All variants have the wild-type cysteine at position 69. Through replacement of the residue at position 1, or position 131, or position 178, flavodoxin variants A001C, D131C, and S178C were generated, respectively. Recombinant and wild-type flavodoxins were expressed in *Escherichia coli* TG2 cells, which grew in Terrific Broth. Each flavodoxin variant was purified according to well-established procedures [31]. Purified proteins have a ratio of absorbance at 280 nm and absorbance at 450 nm (i.e., A280/A450) of about 4.75, demonstrating that all molecules contain FMN. To avoid oxidation of cysteine thiols, dithiothreitol (DTT) was present during protein purification.

The buffer used in all experiments with purified protein was 100 mM potassium pyrophosphate (KPPi), pH 6.0, unless otherwise mentioned.

### Site-specific Labeling of Cys001, Cys131, or of Cys178 with Acceptor in Holoprotein

Acceptor label (i.e., A568; Invitrogen) was added in 2.5-fold molar excess to purified cysteine-pair containing flavodoxin, and after 15 minutes at 22°C, the reaction was stopped by adding 10-fold molar excess of reduced glutathione (Sigma). Labeling during this short period largely prevents dye labeling of the relatively inaccessible Cys69. Subsequently, by using gel filtration with a P6-DG column (Bio-Rad), dye-labeled and non-labeled flavodoxin were separated from unreacted label, and buffer was exchanged to 20 mM Bis-Tris-HCl (Duchefa), pH 6.0. Singly acceptor-labeled protein was separated from non-labeled flavodoxin and from a small fraction of doubly acceptor-labeled protein, using ion exchange chromatography with a MonoQ 5/5 HR column (Pharmacia). Elution was done in the Bis-Tris-HCl buffer mentioned, using a salt gradient ranging from 0 to 1 M KCl.

### Site-specific Labeling of Cys69 with Donor in Unfolded Protein

Singly acceptor labeled flavodoxin was unfolded in 6 M guanidine hydrochloride (GuHCl; Fluka), 100 mM KPPi, pH 7.0, to optimize accessibility of Cys69 for labeling. Subsequent addition of 10-fold molar excess of donor label (i.e., A488; Invitrogen) at 22°C for a period exceeding 60 minutes led to labeling of Cys69. The resulting doubly dye-labeled apoflavodoxin molecules (i.e., d69-a1, d69-a131, or d69-a178) were separated from unreacted label, FMN and GuHCl using gel filtration with a Superdex75 10/30 HR column (Pharmacia). To obtain ‘donor-only’ protein (i.e., d69-apoflavodoxin), wild-type apoflavodoxin (i.e., protein that contains a single cysteine at position 69) was labeled with A488 using the same procedure to label Cys69 of singly-acceptor labeled protein. All dye-labeled apoflavodoxin was stored in 3 M GuHCl at −20°C.

### Denaturant Induced Equilibrium (un)Folding

To determine the concentration of dye-labeled protein stock, absorption spectra of singly and doubly labeled apoflavodoxin were acquired on an HP-8453 diode array spectrophotometer. Label concentrations were determined using absorption coefficients of 71000 M^−1^ cm^−1^ and 91300 M^−1^ cm^−1^ for A488 and A568, respectively.

For each data point in a denaturant-dependent equilibrium folding series of dye-labeled apoflavodoxin, 50 µL of 1.25 µM protein stock in 2 M GuHCl was diluted into 950 µL of the appropriate GuHCl concentration using a Hamilton syringe. Final protein concentration was 62.5 nM. Samples were at equilibrium, since no change in fluorescence was observed after 5 minutes of incubation. For practical reasons, prior to measurements, samples stood for 16 to 24 hrs in the dark at 25°C.

Steady-state fluorescence measurements of denaturant-dependent equilibrium folding at 25°C were done on a Cary Eclipse fluorescence spectrophotometer (Varian). Several combinations of excitation and emission wavelengths were used. Protein fluorescence emission was measured at 330 nm, upon excitation at 280 nm. At this wavelength, both tyrosine and tryptophan residues are excited, but fluorescence emission at 330 nm mainly arises from tryptophans. Donor was excited at 450 nm, to avoid direct excitation of the acceptor, and donor fluorescence emission was measured at 515 nm. Acceptor was excited at 580 nm and acceptor fluorescence emission was measured at 630 nm. Sensitized emission of acceptor fluorescence was measured at 630 nm upon excitation of donor at 450 nm. All fluorescence signals were recorded for 7.125 seconds. Excitation and emission slits were set to a width of 5 nm, except for the measurement of tryptophan fluorescence, where the emission slit was set to 10 nm.

To avoid protein adsorbing to glass surfaces, Tween-20 was added to all solutions to a final concentration of 0.0035% (w/v). This addition does not affect apoflavodoxin, since no change in thermal midpoint of apoflavodoxin unfolding is observed. Refractometry was used to determine the GuHCl concentration in each individual sample [53].

### Time-dependent Fluorescence Anisotropy

Time resolved fluorescence was acquired using the time-correlated single photon counting technique, as described elsewhere [54]. For measurement of donor fluorescence lifetime and anisotropy, excitation was at 450 nm and fluorescence emission was detected using a Schott 512.2 nm (FWHM ∼13 nm) interference filter, in combination with a Schott GG475 (475 nm) long pass filter. For measurement of acceptor fluorescence lifetime and anisotropy, excitation was at 575 nm and fluorescence emission was detected using a Balzers 635 nm (FWHM ∼13 nm) interference filter, in combination with a Schott RG610 (610 nm) long pass filter. Pulse duration was 0.2 ps, pulse energies were at the pJ level and the repetition rate of excitation pulses was 3.8×10^6^ Hz. Samples were kept at 25°C. Decay curves were collected in 4096 channels of a multi-channel analyzer using a channel time spacing of 5.0 ps. Measurements consisted of ten repeated cycles of 10 s duration of parallel (I_||_ (t)) and perpendicularly (I_⊥_ (t)) polarized fluorescence emission. Background fluorescence was measured under the same conditions. For the deconvolution procedure, the dynamic instrumental response function was determined using freshly made solutions of Erythrosine B in H_2_O (*τ* = 80 ps) and pinacyanol in 100% MeOH (*τ* = 6 ps), both with an OD of 0.1 at the wavelengths used for donor and acceptor excitation, respectively.

The total fluorescence decay *I(t) (I(t) = I*_||_ *(t) +2I*_⊥_ *(t))* was analyzed using a sum of discrete exponentials with lifetimes *τ_i_* and amplitudes *α_i_*. The time-dependent fluorescence anisotropy *r(t) (r(t) = (I*_||_ *(t)−I*_⊥_ *(t))/I(t))* was from parallel and perpendicular intensity components [55]. Data analysis was done using TRFA data processor (Scientific Software Technologies Center, Minsk).

## Supporting Information

Figure S1 **In flavodoxin, Cys69 is much less accessible than Cys1, Cys131, and Cys178.**
(DOC)

Figure S2 **Biogel P6DG elution profiles of wild-type and S178C flavodoxin after labeling of these proteins with A568.**
(DOC)

Figure S3 Figure S3A: Doubly labeled apoflavodoxin has equimolar ratio of donor to acceptor. Figure S3B: Spectral overlap exists between tryptophans and A488 and between tryptophans and A568.
(DOC)

Figure S4 **Examples of experimental fluorescence anisotropy decay curves (grey lines) and associated bi-exponential fits (black lines) obtained for A568 of doubly dye-labeled apoflavodoxins and for A488 of d69-apoflavodoxin.**
(DOC)
