# Supplementary material for: Illuminating the Off-Pathway Nature of the Molten Globule Folding Intermediate of an α-β Parallel Protein
Source: PLoS One. 2012 Sep 21;7(9):e45746. doi: 10.1371/journal.pone.0045746 (PMC3448718; doi:10.1371/journal.pone.0045746)

**Figure S1.** **In flavodoxin, Cys69 is much less accessible than Cys1, Cys131, and Cys178.** The cartoon model shows the surface of flavodoxin in a semi-transparent fashion, with Cys69 in blue, and the other cysteines in red. The FMN cofactor is shown in yellow. The cartoon model and the cysteines were generated with PyMOL (Schrödinger, LLC, Palo Alto, Ca, USA) using the crystal structure of *A. vinelandii* flavodoxin (pdb ID 1YOB ).


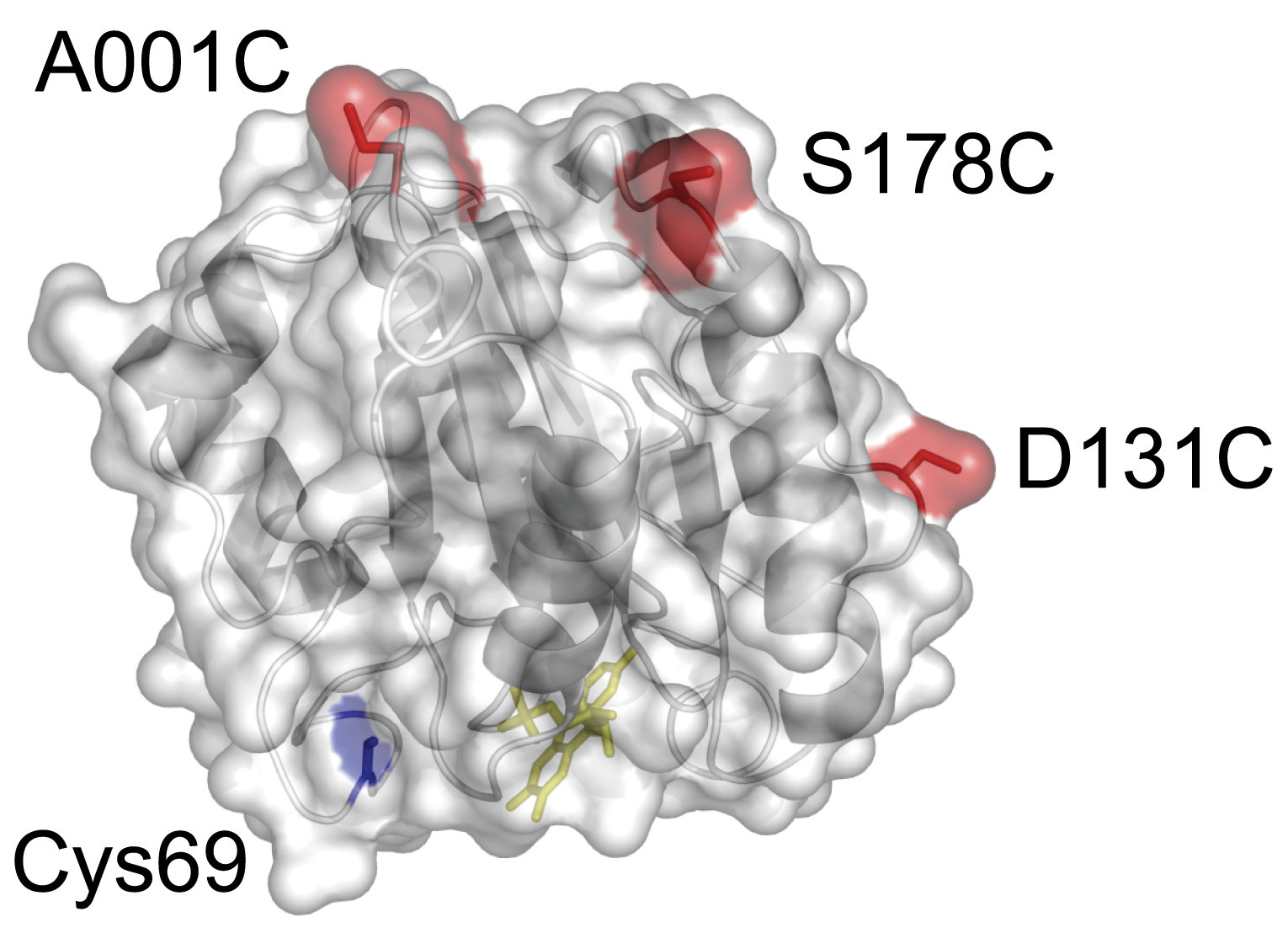

Supplement: Figure S1 — In flavodoxin, Cys69 is much less accessible than Cys1, Cys131, and Cys178. (DOC) [file pone.0045746.s001.doc]
