# Supplementary material for: Illuminating the Off-Pathway Nature of the Molten Globule Folding Intermediate of an α-β Parallel Protein
Source: PLoS One. 2012 Sep 21;7(9):e45746. doi: 10.1371/journal.pone.0045746 (PMC3448718; doi:10.1371/journal.pone.0045746)

**Figure S2: Biogel P6DG elution profiles of wild-type and S178C flavodoxin after labeling of these proteins with A568.** (A) Flavodoxin variant S178C, which contains cysteines at positions 69 and 178, respectively. (B) Wild-type flavodoxin, which contains only cysteine at position 69. Solid lines represent absorption at 578 nm (i.e., due to presence of A568), dashed lines represent absorption at 450 nm (i.e., due to presence of FMN) and dotted lines represent absorption at 280 nm (i.e., due to tryptophan, FMN and A568). Labeling is done as described in Materials and Methods, using identical concentrations of S178C and wild-type flavodoxin. Protein elutes at about 3.5 ml of elution volume, whereas free label elutes at larger elution volumes. The data clearly show that under the given experimental conditions hardly any labeling of Cys69 takes place.


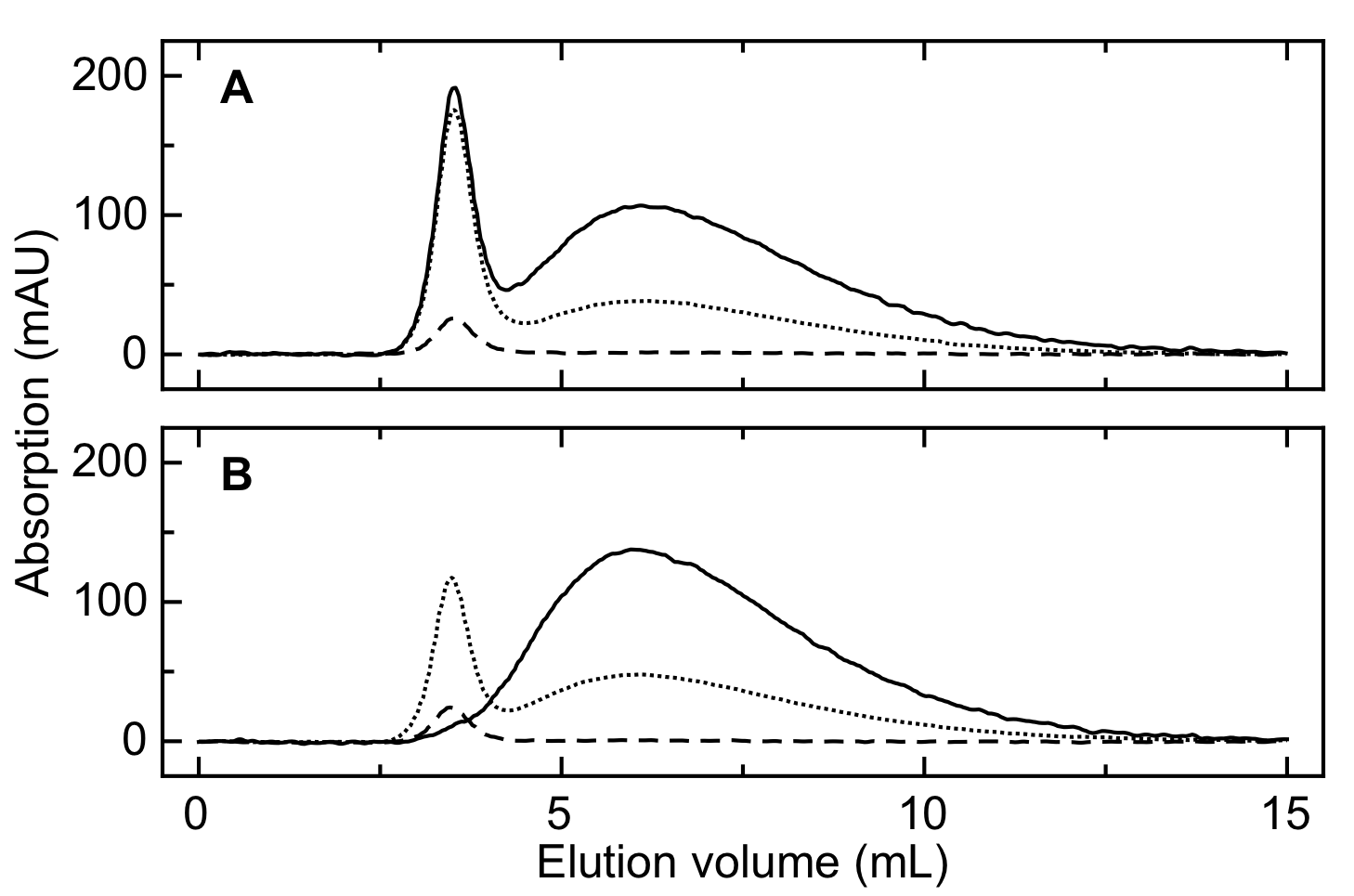

Supplement: Figure S2 — Biogel P6DG elution profiles of wild-type and S178C flavodoxin after labeling of these proteins with A568. (DOC) [file pone.0045746.s002.doc]
