# Supplementary material for: Illuminating the Off-Pathway Nature of the Molten Globule Folding Intermediate of an α-β Parallel Protein
Source: PLoS One. 2012 Sep 21;7(9):e45746. doi: 10.1371/journal.pone.0045746 (PMC3448718; doi:10.1371/journal.pone.0045746)

**Figure S3.** **(A) Doubly labeled apoflavodoxin has equimolar ratio of donor to acceptor.** Normalized absorption spectra of d69-a1 (blue), d69-a131 (green) and d69-a178 (orange) are shown. Based on absorption coefficients of 71000 M^-1^ cm^-1^ and 91300 M^-1^ cm^-1^ for A488 and A568, respectively, the ratio of donor to acceptor is calculated to be equimolar. Dye-labeled proteins are in 3 M GuHCl.


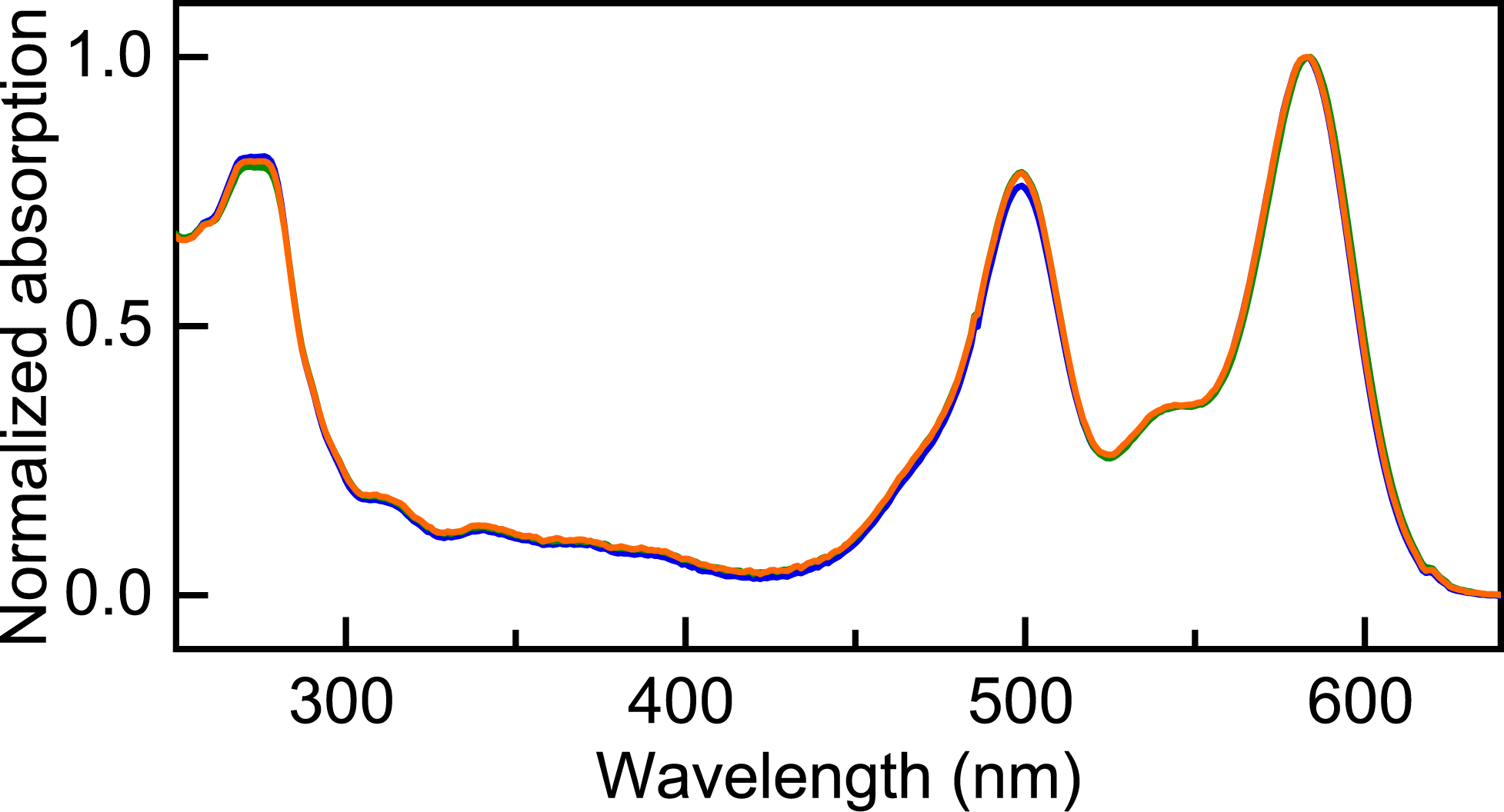


**(B) Spectral overlap exists between tryptophans and A488 and between tryptophans and A568.** Spectra are normalized to their respective maxima (i.e., 499 nm for A488, 579 nm for A568, and 331 nm for apoflavodoxin). Using these spectra, with ε_max-A488_ = 71000 M^-1^cm^-1^ and ε_max-A568_ = 91300 M^-1^cm^-1^, together with equation 9, we calculate considerable spectral overlap integrals between tryptophans and A488 and between tryptophans and A568, respectively (i.e., J_Trp-A488_ = 2.75 10^14^ nm^4^ M^-1^ cm^-1^ and J_Trp-A568_ = 1.95 10^14^ nm^4^ M^-1^ cm^-1^). Using equation 4, the corresponding *R_0_*-values are calculated to be ~ 26 Å and ~ 27 Å, respectively (with *n* = 1.5, *κ*^2^ = ⅔, *Q_D_* = 0.2). Table 1 shows that distances between tryptophans of apoflavodoxin and attached dye labels are well within these Förster distances. Thus, Förster resonance energy transfer occurs from tryptophan residues to either of the attached fluorophores.


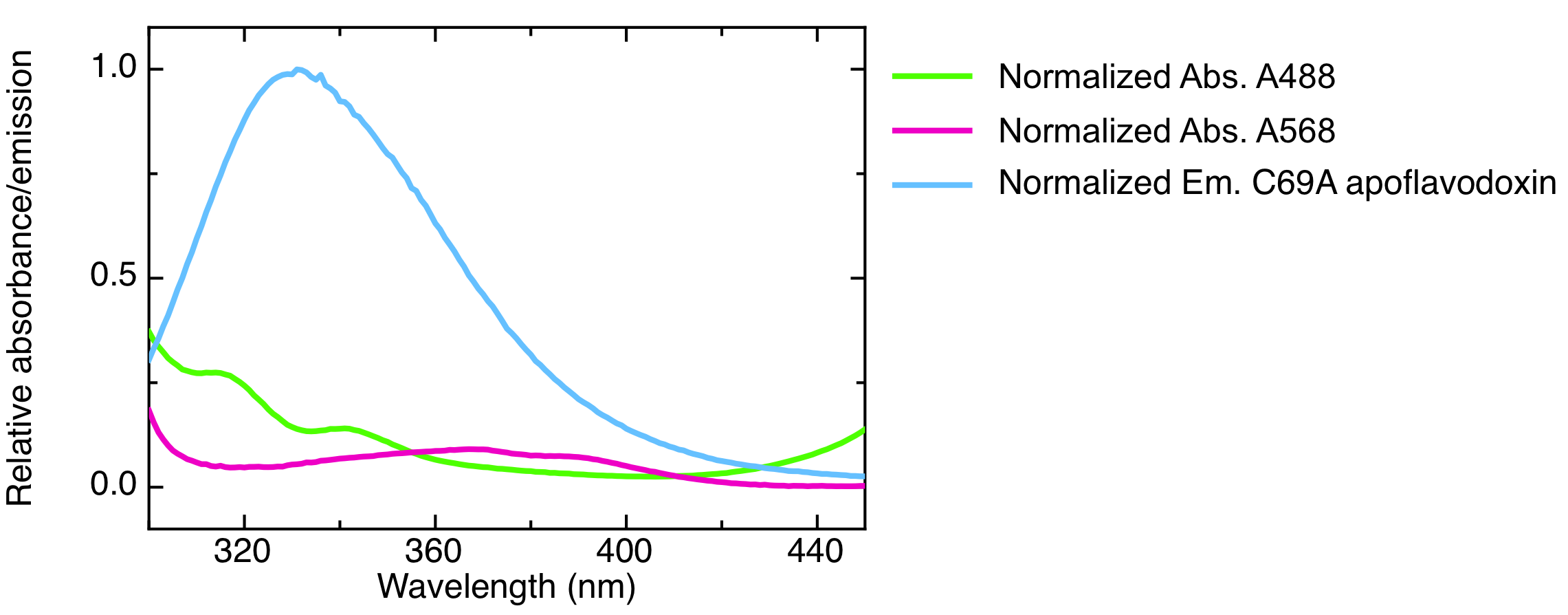

Supplement: Figure S3 — A: Doubly labeled apoflavodoxin has equimolar ratio of donor to acceptor. Figure S3B: Spectral overlap exists between tryptophans and A488 and between tryptophans and A568. (DOC) [file pone.0045746.s003.doc]
