# Supplementary material for: Illuminating the Off-Pathway Nature of the Molten Globule Folding Intermediate of an α-β Parallel Protein
Source: PLoS One. 2012 Sep 21;7(9):e45746. doi: 10.1371/journal.pone.0045746 (PMC3448718; doi:10.1371/journal.pone.0045746)
Supplement: Figure S4 — Examples of experimental fluorescence anisotropy decay curves (grey lines) and associated bi-exponential fits (black lines) obtained for A568 of doubly dye-labeled apoflavodoxins and for A488 of d69-apoflavodoxin. (DOC) [file pone.0045746.s004.doc]

**Figure S4. Examples of experimental fluorescence anisotropy decay curves (grey lines) and associated bi-exponential fits (black lines) obtained for A568 of doubly dye-labeled apoflavodoxins and for A488 of d69-apoflavodoxin.** Shown are anisotropy decays obtained for protein in 0 M, 1.3 M and 5 M guanidine hydrochloride, respectively. Weighted residuals are shown to illustrate the quality of the fits.

**
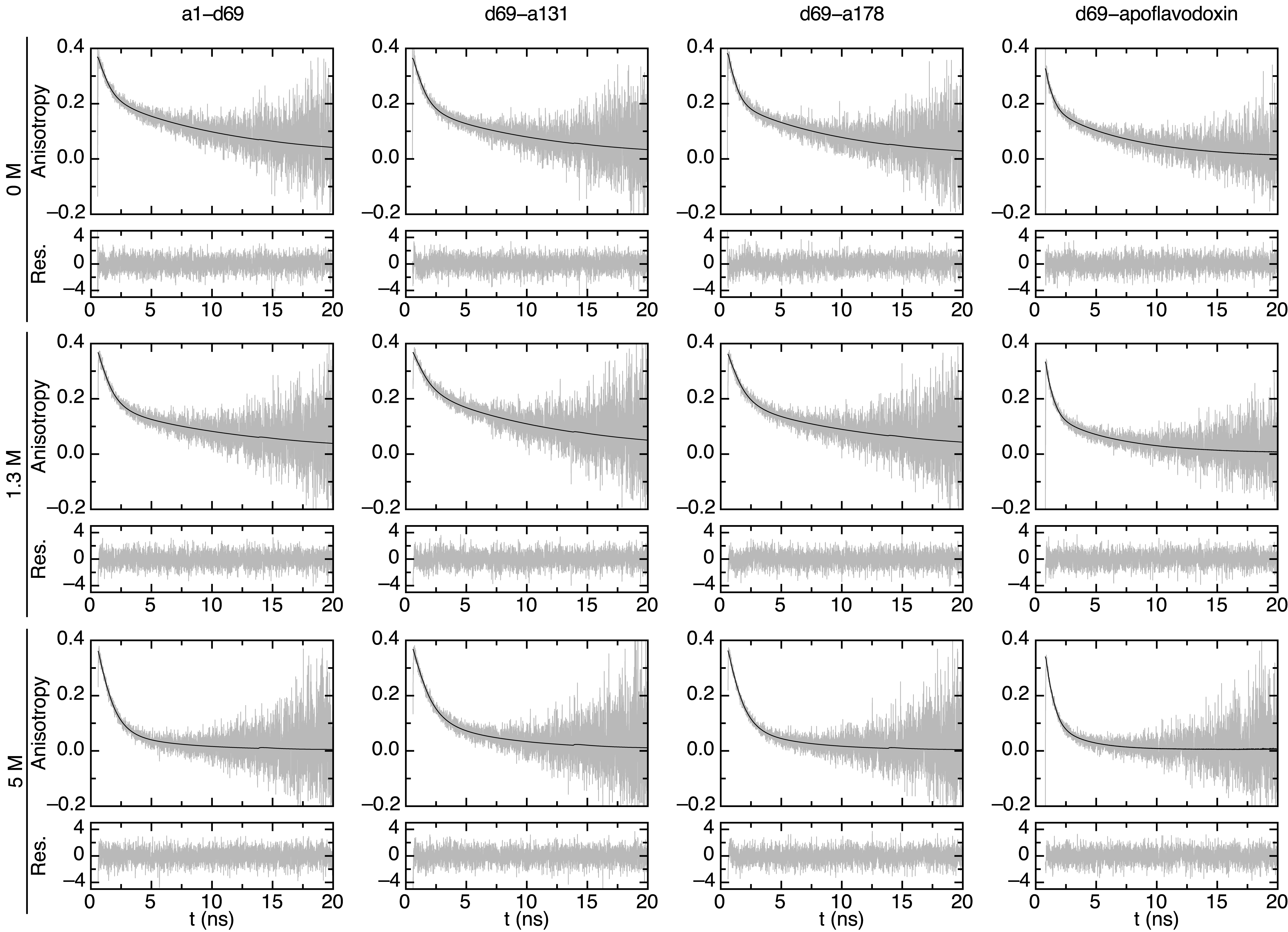
**
